# Trends in Urban Wild Meat Trade of Chelonians (Turtles and Tortoises) in the Peruvian Amazon

**DOI:** 10.3390/ani14223205

**Published:** 2024-11-08

**Authors:** Pedro Mayor, Richard Bodmer, Kelly Moya, Samantha Solis, Maire Kirkland, Pedro Perez-Peña, Tula Fang, Martí Orta-Martínez

**Affiliations:** 1 Departament de Sanitat i d’Anatomia Animals, Facultat de Veterinària, Universitat Autònoma de Barcelona (UAB), 08193 Barcelona, Spain; 2 Museo de Culturas Indígenas Amazónicas, Fundamazonia, Iquitos 16006, Peru; r.bodmer@kent.ac.uk (R.B.); tulafang@gmail.com (T.F.); 3 ComFauna, Comunidad de Manejo de Fauna Silvestre en la Amazonía y en Latinoamérica, Iquitos 16006, Peru; pperez@iiap.gob.pe; 4 Durrell Institute of Conservation and Ecology (DICE), School of Anthropology and Conservation, University of Kent, Canterbury CT2 7NR, UK; 5 Faculty of Biology, Universidad Nacional de la Amazonia Peruana (UNAP), Iquitos 16001, Peru; kemova_07@hotmail.com (K.M.); ssolis703@gmail.com (S.S.); 6 British Trust for Ornithology, Thetford IP24 2PU, UK; maire.kirkland@hotmail.co.uk; 7 Instituto de Investigaciones de la Amazonía Peruana (IIAP), Iquitos 16001, Peru; 8 Department of Evolutionary Biology, Ecology and Environmental Sciences, University of Barcelona, Catalonia, 08028 Barcelona, Spain; marti.orta@ub.edu

**Keywords:** tortoises, river turtles, *Podocnemis*, *Chelonoidis*, eggs, wild meat, urban markets

## Abstract

Culturally, chelonians are important sources of nutrition and income for rural and urban people in tropical rainforests, but urban trade can cause declines in wild populations. This study analyses the urban chelonian trade and its trends to better understand the impact on free-living populations in the Northern Peruvian Amazon. We studied the urban trade of wild chelonians by conducting 526 days of participant observation and structured questionnaires with the main chelonian vendors in the wet markets of Iquitos between 2006 and 2018. The trade of chelonians decreased by −161.6%, from 22,694 individuals in 2006/07 to 8657 individuals in 2017/18. *Chelonoidis denticulatus* was the species sold the most (86.3%), followed by *Podocnemis unifilis* (13.6%). *Podocnemis expansa* was only sold in 2006/07. River turtle sales increased in the dry season, while sales of *Chelonoidis denticulatus* decreased. Turtles were the most expensive meat sold in urban markets: 49.7% more expensive than the most frequently consumed fish, *Prochilodus nigricans*, and 48.4% more expensive than poultry. Turtles represented only 0.19% (SD 0.23) of the index of domestic meat and fish consumption per capita. River turtle eggs are sold by a different set of vendors, and in 2017/18, 570,229 eggs were traded in the market of Belén in Iquitos, equivalent to an approximate lay of 10,418 *P. unifilis* and 1178 *P. expansa* females. The high magnitude of the trade of river turtle eggs together with the reduction in their meat trade suggests a prioritization of egg sales, which should improve the conservation of adults, since meat sales can be detrimental to chelonian populations.

## Simple Summary

Turtles provide the rural and urban populations of tropical forests with food and income. We studied one of the largest urban wild meat markets in the Amazon between 2006 and 2018. Turtle meat was the most expensive animal protein, being 50% more costly than the most common fish, *Prochilodus nigricans*, and 48% more costly than poultry meat. Most of the turtle meat was from *Chelonoidis denticulatus* (86.3%) and *Podocnemis unifilis* (13.6%). Eggs from *P. unifilis* and *P. expansa* were also sold.

## 1. Introduction

Globally, chelonians are classified as one of the most threatened taxonomic groups, with many species suffering population decline or local extinction [1,2,3]. In tropical forests, chelonian meat and eggs are an important source of subsistence protein for rural people [4,5]. However, overharvesting for meat, eggs, oil, and the pet trade has contributed to the global decline in chelonians.

Chelonians have been harvested for food and oil production for centuries in the Amazon basin. River turtles were one of the most intensely harvested species in the Amazon during the 19th and 20th centuries, and populations were decimated [6,7,8,9]. People harvested adults, especially females, when they laid eggs on beaches in the dry season, and at the same time collected the eggs, affecting all life stages and removing reproductive females [6,10].

In the Amazon, the integration of rural people into the market economy increased the supply and demand for chelonian products in urban centers [11]. Chelonians are an important part of the wild meat trade and, in the Central and Western Amazon, they make up 19.8% of wild meat consumption [4,12,13]. Wild meat is sold in wet markets, and studies have shown a diversity of traded species, with the most common groups being tortoise and river turtle meat, river turtle eggs, caiman, game birds, ungulates, and large rodents [4,14,15,16,17]. For instance, the long-term monitoring of the wild meat trade in the wet markets of Iquitos, the largest city in the Peruvian Amazon, showed that, although the amount of wild mammal meat biomass sold from 1973 to 2018 increased significantly at a rate of 6.4 t/year, reaching maximum trade rates of 442 tn in 2017/18, the contribution of wild mammal meat to overall meat consumed was constantly 1–2% [15].

The magnitude, impact and sustainability of chelonian trade needs to be better understood, since river turtles and tortoises are an important resource for Amazonian people and, more recently, have been used for cash income through the pet trade [12,18]. In the late 20th century, head-starting programs began throughout the Amazon, and turtle populations have largely recovered in areas that have these programs. In the Peruvian Region of Loreto, a large-scale head-starting program spanning the Pacaya-Samiria National Reserve and other areas over the past two decades has been important for the recovery of river turtles, and the program has released over one million hatchlings [8,19]. In contrast, tortoises are an elusive species that are the preferred meat for local people because they can be kept alive until eaten [4,17]; however, there are no head-starting programs for these species [20].

Chelonian trade in urban markets can be used as an indicator of harvest levels over a broad geographical region and can be used to assess the impacts on and changes in harvests caused by rural people [5,14]. Chelonians meat and eggs are openly traded in the wet markets of Iquitos, which allowed us to study (1) the species of chelonians being sold, (2) the products they are selling from these species, (3) the volume traded, (4) the trend of this market over the last two decades, and (5) prices of chelonian meat compared to domestic meat, wild mammal meat, and fish. The study of the urban trade of chelonians provides key information on the importance of chelonians for the dietary animal protein intake of the urban population, as well as a better understanding of the sustainability and conservation of chelonians in the Amazon.

## 2. Materials and Methods

### 2.1. Study Area

Loreto is the largest (386,851 km^2^) region in Peru [21], with a population of 1055.182 inhabitants (2.4 inhabitants/km^2^). The Indigenous population of 106,900 (31.8%) is grouped in 705 recognized Indigenous communities [21]. The two major landscapes in Loreto are upland forests (*terra firme*) and seasonally flooded forests (*várzea*), which have different but overlapping resources and Indigenous cultures, and have differences in their resource management and economies [22]. The communities living in flooded forests have divergent livelihood strategies to cope with annual fluctuations in water levels [23].

**Figure 1:**
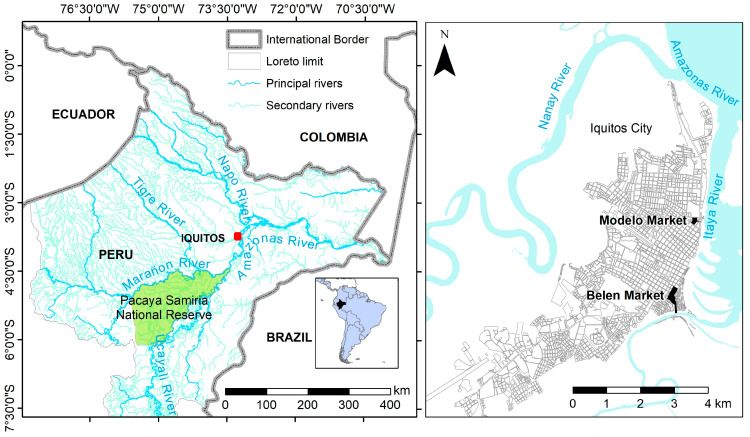
Map of the Loreto region (on the **left**) and the city of Iquitos (on the **right**). The map of Loreto shows Iquitos (red dot), the main hunting basins for *Cheloinidis denticulatus* and the Pacaya-Samiria Natural Reserve, the most important area for collecting river turtles. The map of Iquitos includes the studied markets of Belén and Modelo.

Our study was conducted in the two largest wet markets of Iquitos, the third largest city in the Amazon and the largest in the Peruvian Amazon (Figure 1), with a population of 486,338 inhabitants in 2018 [Gobierno Regional de Loreto-GOREL-, personal communication].

Iquitos has two large open wet markets: (i) Belén, which is the most important market for fish, wild meat, and turtle meat and eggs in Iquitos; and (ii) Modelo, which is an important, but secondary urban market [15]. These two markets offer a good proxy of the total river turtle and tortoise meat consumption in Iquitos [15]. In this region, there are few roads, and products are mainly transported from rural areas to Iquitos by boat. Both markets offer many types of non-timber forest products, from traditional medicines and pets to fresh fruits and vegetables.

In Iquitos, turtle and tortoise meat has been sold traditionally for many years, although the Peruvian Ministry of Agriculture, through laws enacted in 1976 (number 21,147), 2000 (number 27,308), and 2011 (number 29,763), only authorizes the sale of wild meat from areas with planned sustainable wildlife management [15]. However, weak enforcement means wild meat is sold openly in these markets, making it relatively easy to monitor.

Tortoises are captured by local hunters and river turtles are captured by fishermen. Intermediary merchants, so-called “*regatones*”, buy or exchange products to acquire live turtles and transport them to the main ports of Iquitos, where they are sold to a limited number of urban market vendors. These vendors buy large quantities of chelonians that they usually keep in the courtyards of houses near the market. Daily, vendors will butcher a few individuals to sell. When the stock begins to decrease, they will purchase a new batch of individuals.

### 2.2. Data Collection

In Belén and Modelo markets, 526 day surveys were conducted during the periods September 2006–August 2007, November 2013–October 2014 (only in the market of Belén), and September 2017–August 2018 (Table 1). The surveys were carried out on 48.0% of the days, and the surveys were distributed evenly throughout the year. On average, each market was surveyed 9.9 days per month (SD 4.9, range 2.7–15.0), which is above the minimum sampling effort of two surveys/month recommended to obtain proper accuracy and precision in wild meat sale and price estimations [24].

The survey methods were the same in all the studied periods. To engage vendors and encourage participation, preliminary market visits were conducted during the two months before the start of each survey period and, during these visits, chelonian vendors were identified. We followed the ethical human-subject guidelines of Buppert and McKeehan [25]. Interviewees participated voluntarily, could leave the study at any time, and were informed of the aims of the study and that all data would be anonymous. All (100%) regular vendors (those who sold more than one day per week) and 90–95% of occasional vendors of wild meat agreed to participate.

To gather information on the individual chelonians sold and the price of turtle species, we combined interviews with participant observation, which was conducted from market opening time to closing time (6:00 to 12:00 a.m.). We recorded the date, species, selling price per individual or portion, and number of individuals brought by vendors at 6:00 a.m., including the individuals displayed on the stall and stored indoors, and the individuals left at 12:00 a.m. The number of individuals sold was calculated as the difference between the individuals on sale at opening time and the individuals left at closing time. This helped avoid the double counting of individuals brought out again the next day.

In 2017/18, we used parallel interviews to assess the consistency of the information obtained, which is a concern of wildlife market studies in the tropics. Two additional questionnaires were used: (1) for one year, our most confident chelonian vendor (“key informant”) registered all chelonian purchases from intermediaries, including date, number of individuals, species, and purchase and sale price; and (2) a unique semi-structured questionnaire was applied to the four most frequent vendors of chelonians to collect information on species, price, availability of turtles, and descriptions of the vendor and the customers.

Additionally, participant observation was used to monitor turtle egg vendors and record the number of vendors and the quantity of eggs sold at Belen market.

### 2.3. Estimations of Consumption and Prices

The amount of fresh meat traded per species was estimated by multiplying the number of individuals by the body weight estimates after evisceration following the calculation proposed by Bardales-García et al. [26].

We calculated the percentage change in individuals between two consecutive periods following the formula [% Change individuals = (year1 − year2)/year2], where year1 and year2 are the number of turtles sold in the two periods studied.

The money spent by vendors to obtain chelonians from intermediaries or hunters and the daily selling price are presented in USD per individual or portion of turtle species. We used the exchange rate from 4 October 2018 (PEN 3.32 = USD 1.00) for 2017/18. Official prices and information on the production of all domestic meat were obtained from GOREL in 2018 [GOREL, personal communication]. In addition, during the survey period of 2017/18, local vendors were interviewed to calculate the average daily price per kg of meat for all domestic species (poultry pork, beef, sheep, and goat), and the most frequently consumed fish (i.e., *Prochilodus nigricans*).

The index of domestic meat and fish consumption per capita (ICPC in Spanish) was calculated by dividing the daily amount of meat produced (for domestic species) or fish landed in Iquitos by the number of inhabitants in Iquitos from 2006 to 2018 [GOREL, personal communication]. A similar index was calculated for chelonians to estimate the relative importance of chelonians for the urban population.

### 2.4. Statistical Analyses

To study trends in chelonian trade, paired *t*-tests were used to compare the number of monthly vendors between the surveys conducted in 2006/07 and 2017/18. To study the association between the number of vendors and the number of chelonians traded per species, Pearson’s correlation was used. To study chelonian trade seasonality, Pearson’s correlation was used to determine the relationship between river water levels, chelonian trade prices, and volume in 2017/18. Monthly data on Amazon river water levels were obtained from the Tamshiyacu hydrological station (76°33′00′′ W, 07°03′00′′ S) of the Peruvian National Meteorology and Hydrology Service [27]. Statistical analyses were performed using R-Studio version 0.98.1062 (RStudio Inc., Vienna, Austria).

## 3. Results

There was a total of 111 vendors selling chelonian meat during the three survey periods, averaging 3.93 (SD 2.42) vendors on each survey day (3.55 [SD 2.56] vendors in 2006/07; 4.26 [SD 1.44] in 2013/14; and 4.76 [SD 2.01] in 2017/18). We identified 31 (27.9%) regular and 80 (82.1%) occasional vendors.

### 3.1. Chelonian Trade and Consumption

Four different chelonian species make up the majority of sales in the markets of Iquitos: *Chelonoidis denticulatus* (yellow-footed tortoise), *Podocnemis unifilis* (yellow-spotted river turtle), *Podocnemis expansa* (giant Amazon river turtle), and *Podocnemis sextuberculata* (six-tubercled Amazon river turtle) (Table 1).

The species sold most frequently in all the studied periods were *C. denticulatus* (76.8% to 86.3% of the total number of chelonians sold) and *P. unifilis* (13.65% to 17.18%). *P. expansa* and *P. sextuberculata* were only sold in 2006/07 (5.85% and 0.15%, respectively). The overall number of chelonians sold decreased −161.1% between 2006/07 and 2017/18, from 22,694 individuals/year (89.91 tones/year) to 8656.8 individuals/year (26.56 tones/year) (Table 1). *Podocnemis unifilis* trade decreased −230.03% and *C. denticulatus* trade decreased −133.19%, and *P. expansa* and *P. sextuberculata* were no longer sold in 2017/18.

**Figure 2:**
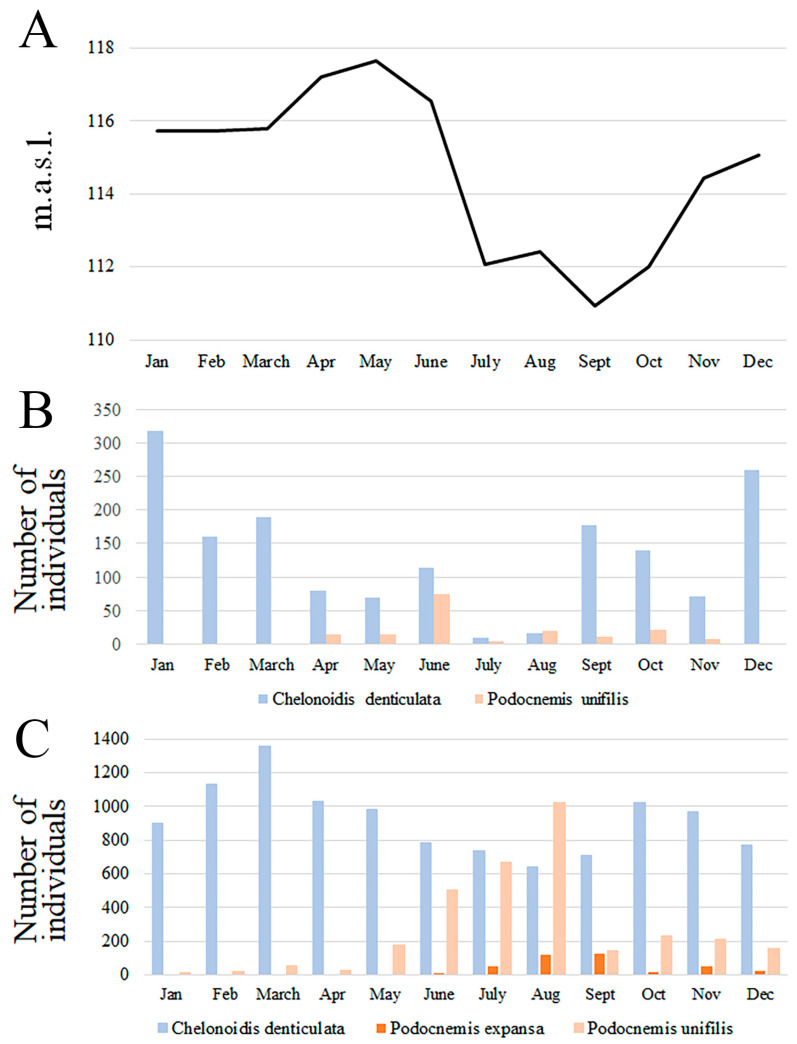
Seasonality of the urban trade of chelonians in Iquitos: (**A**) the river level (meters above sea level, m.a.s.l.) of the Amazon River provided by the Peruvian National Meteorology and Hydrology Service [27]; (**B**) number of individuals of *Chelonoidis denticulatus* and *Podocnemis unifilis* purchased by fixed vendors from intermediaries in local markets in Iquitos in 2017/18; and (**C**) number of individuals of *Chelonoidis denticulatus*, *Podocnemis unifilis*, and *P. expansa* sold in urban markets in Iquitos in 2006 and 2018. All the individuals sold each month have been grouped by adding the two 12-month surveys.

The sale of river turtles occurred predominantly in the dry season (74.1% of *P. expansa* individuals were sold between July and September, and 68.0% of *P. unifilis* between June and August), and concurred with the lower sales of *C. denticulata* (Figure 1, Supplementary Table S1). Only the sales of *P. expansa* showed a correlation with the Amazon River water levels (r = −0.790, *p* = 0.022). There was no correlation with other river water levels or other chelonian species (*C. denticulata*, *p* = 0.1124; *P. unifilis*, *p* = 0.1458; *P. sextuberculata*, *p* = 0.2367). This seasonal sale pattern was similar to the pattern of purchases from intermediaries estimated from general monitoring and from our ‘key informant’ (Figure 2, Supplementary Tables S2 and S3; Supplementary Figure S1).

The average ICPC (index of consumption per capita) for animal protein in Iquitos was 193.2 (SD 58.0) g/inhabitant/day during the study period (2006–2018). The ICPC for chelonians remained low over the whole study period (0.29 [SD 0.30] g/inhabitant/day), representing only 0.19% (SD 0.23) of the total ICPC, and decreasing from 0.64 g/inhabitant/day (0.46%) in 2006/07 to 0.15 g/inhabitant/day (0.05%) in 2017/18 (Table 2). This was despite the human population in Iquitos growing from 386,037 to 486,338 inhabitants between 2006 and 2018. On average, each inhabitant of Iquitos consumed one turtle every 33 years: an annual consumption of 0.0178 chelonians per inhabitant. In contrast, poultry contributed to 72.95% (SD 8.70) of the ICPC, fish 18.11% (SD 10.08), porcine meat 5.60% (SD 1.96), bovine meat 1.33 (SD 0.37), and wild meat 1.26% (SD 0.35). We observed a non-significant correlation between price and consumption rate (*p* = 0.1438).

**Figure 3:**
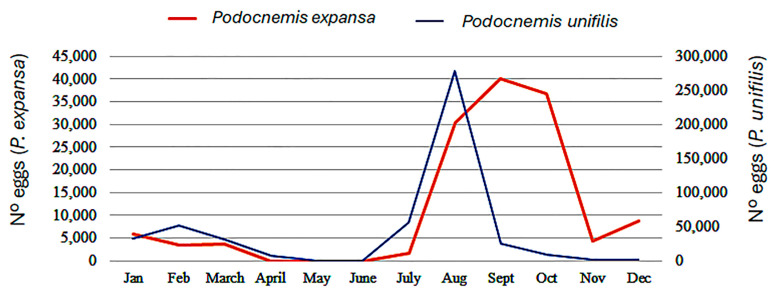
Seasonality of the number of eggs sold in wet markets of Iquitos during 2017 and 2018.

There were 26 vendors of river turtle eggs, with 14 (53.84%) regular and 12 (44.16%) opportunistic sellers in the 12-month study period of 2017/18 in the wet markets of Iquitos. The sale of eggs and meat was undertaken by different vendors. The annual estimated egg sales comprised 632,283 eggs, composed of 497,394 (78.67%) *P. unifilis* eggs and 134,889 (21.33%) *P. expansa* eggs. No trade of tortoise eggs was reported. The number of eggs sold daily was 249.6 (SD 170.1), with 271.8 (SD 178.4) of *P. unifilis* and 187.2 (SD 124.6) of *P. expansa*. According to the preservation methods, 82.37% were sold cooked, 16.75% salted, and 0.88% fresh. The seasonal pattern shows that the sale of *P. unifilis* eggs was concentrated in August (278,082 eggs, 55.91%) and the sale of *P. expansa* eggs was concentrated in the period between August and October (107,032 eggs, 79.35%) (Figure 3).

### 3.2. Prices and Economic Profit of Chelonians Traded

In 2017/18, the mean consumer purchase price of chelonians was USD 18.77/individual (SD 4.51), while the mean vendor purchase price was USD 13.74/individual (SD 3.30) (Table 3), generating a gross profit per individual sold of USD 5.03 (26.78%). Consumer purchase price was USD 21.25/individual (SD 5.56) for *P. unifilis* and USD 18.27/individual (SD 3.90) for *C. denticulatus*, resulting in an average gross profit of USD 9.93/individual (46.74%) and USD 3.67/individual (20.11%), respectively. Chelonians were usually sold in anatomical portions, with the most frequently sold portions being the hind and forelimbs, liver, and carapace (Table 4).

Chelonian meat was the most expensive meat sold in the urban markets of Iquitos when compared to domestic and wild meat. Chelonian meat was 49.67% more expensive than the most frequently consumed fish, *Prochilodus nigricans*, 48.36% more expensive than poultry, 28.29% more expensive than pork, 28.29% more expensive than beef, and 26.48 more expensive than mammalian wild meat (Table 5).

Vendors spent USD 122,460/year to obtain chelonian meat from intermediaries and hunters in the 2017/18 study period, and meat consumers spent USD 161,657/year on chelonian meat in the markets of Iquitos. The overall gross annual profit was USD 39,197/year, including USD 27,462/year (70.06%) for *C. denticulatus* and USD 11,734/year (29.93%) for *P. unifilis*.

The average price for each egg sold to retailers was USD 0.45 (SD 0.14) in 2017/18, and USD 28.02 (SD 3.18) for the purchase of 100 eggs. The sale price changed depending on the species and the preservation method (Supplementary Table S4). Consumers of turtle eggs spent USD 284,067, including USD 222,272 (78.25%) for eggs of *P. unifilis* and USD 61,794 (21.75%) for *P. expansa*. Since we did not obtain the purchase price of eggs from intermediaries, the overall gross profit from the commercialization of eggs was not estimated.

### 3.3. Vendors’ Performance and Profiles

**Figure 4:**
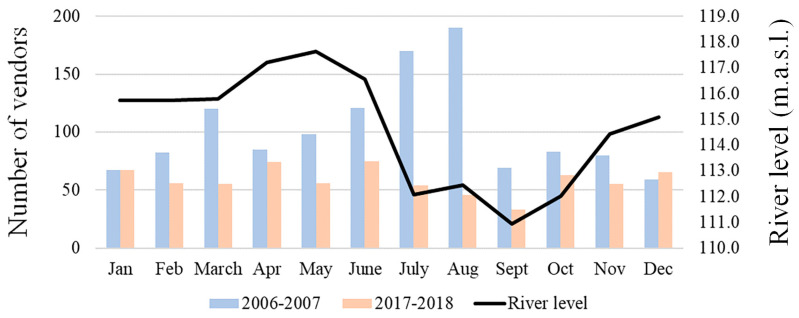
Number of vendors identified per month in the urban markets of Iquitos in the surveys of the years 2006/07 and 2017/18, and the Amazon River level (m.a.s.l.) during 2017/18, provided by the Peruvian National Meteorology and Hydrology Service [27].

In 2006/07, the number of vendors increased in July–August, during the dry season, compared with the other months (t = 3.3523, df = 11, *p* = 0.0064; Figure 4). In this period, we found a correlation between the number of vendors and the number of *P. unifilis* (r = 0.9101, *p* < 0.00001) and *P. expansa* (r = 0.8343, *p* > 0.00001) sold, but not with *C. denticulata* (r = −0.03904, *p* = 0.9041). In 2017/18, we did not observe any seasonal variations in the number of vendors. There was no trade of *P. expansa*, and the correlation between the number of vendors and the number of *P. unifilis* sold became non-significant (r = 0.1595, *p* = 0.6205), while the relationship with *C. denticulata* showed a negative trend (r = −0.7035, *p* = 0.1070).

The single complementary questionnaire used in 2017/2018 to the four main vendors and the records of our ‘key informant’ were used to verify the information between methodologies and provide additional information on the profiles of the vendors. The four main (4/4) vendors were women with a mean age of 64.75 (SD 5.19 years), and they were all born in Iquitos.

The four vendors bought chelonians from intermediaries and purchased chelonians every 3.50 (SD 0.60) days, with a daily sale to consumers of 3.50 (SD 1.73) individuals. Likewise, our ‘key informant’ purchased chelonians every 4.25 days and sold 3.78 individuals per day. Only one vendor bought consistently from the same intermediary, and the others used different intermediaries depending on the price and availability of chelonians.

All four vendors reported trading *C. denticulatus*, and the trade was most frequent between September and April and decreased between May and August. The main provenances of *C. denticulatus* were the basins of the Marañón (4/4), Tigre (3/4), Napo (3/4), and Ucayali (3/4) Rivers (Figure 1). All four vendors characterized customers as women (62.5%) with an age of 46.2 (SD 13.7) years old. Three defined them as poor people, although all vendors reported the occasional sale to wealthier people and small restaurants.

## 4. Discussion

Studies on chelonian trade and consumption have shown the magnitude of this trade throughout the Amazon basin [4,28,29]. During the period 1987–1996, Peres and Dolman [29] estimated an annual consumption of between 504,876 and 1,237,834 turtles in the entire Brazilian Amazon. Despite the declaration of illegality in the Brazilian legislation of 1967 (federal laws 5197/1967 and 9605/1998), studies show that the consumption of river turtles is common [30], with 100% consumption in households in rural areas and small cities in 2006 [28], and 91.2% consumption in households in Manaus, a large Amazonian city, in the late 1990s [31]. The average consumption of turtles was higher in rural areas (21.65 turtles per household) than in urban areas (11.26 turtles per household), and included mainly the yellow-spotted river turtle (*P. unifilis*) and the six-tubercled river turtle (*P. sextuberculata*). Other studies on the Central Amazon based on consumer surveys reported an annual consumption between 0.235 chelonians per inhabitant [12] and 11.26 chelonians per household [28] in small cities, and 2.98 chelonians per household in rural communities [4]. Comparatively, chelonians’ per capita consumption in Iquitos was between 3 and 25 times lower than in the above-mentioned studies in the Central Amazon.

In the Peruvian Amazon, fish and wild meat, including chelonians, are traditional sources of dietary animal protein for rural people and are widely sold in urban markets [14]. However, between 2001 and 2014, poultry production grew by 284% and represented 76% of total daily meat consumption [15], with poultry prices similar to fish, but considerably cheaper than wild mammals (−29.7%) and chelonians (−48.3%). Although the increase in poultry consumption, mainly due to increased production and lower prices, has influenced the wild meat trade [15], other factors might affect the chelonian trade in Iquitos. Chelonian meat was the most expensive animal protein sold in the markets of Iquitos and its contribution to total protein consumption per capita was very small, unlike in the Brazilian Amazon, where the comparative price of turtles is low [32]. In Iquitos, 0.0178 chelonians were consumed annually per inhabitant in 2017/18 and, in the same period, the yearly consumption of chelonian meat was 26.6 tons, which was only 6% of the 442 tons of wild mammals sold [15].

The trade of chelonian meat as a source of urban animal protein decreased for all species over the 12-year period from 2006 to 2018 in the wet markets of Iquitos. We report an annual trade of 8656 chelonians in 2017/18, which shows an impressive reduction from the annual trade of 22,694 individuals in 2006/07. The chelonian per capita consumption decreased from 0.64 g/inhabitant/day (0.46%) to 0.15 g/inhabitant/day (0.05%). The reduction in chelonian trade was observed in all species (−161.1%), with a decrease in the trade of *C. denticulatus* (−133.19%) and *P. unifilis* (−230.03%) and no sales of *P. expansa* or *P. sextuberculata* as a source of meat in 2018.

Between 2007 and 2018, we observed a reduction of more than half in the *C. denticulata* trade, and a reduction of −170% in overall chelonians traded, while the wild mammal meat trade increased +136% during the same period. As a result, in the Iquitos markets, *C. denticulata* was the third most traded species behind *Pecari tajacu*, *Tayassu pecari*, and *Cuniculus paca*, through direct calculations of biomass [15] and through surveys [17]. Over the same period, an increase in the sale of *P. unifilis* and *P. expansa* eggs was observed. The relation between decreasing meat sales and increasing egg sales in the markets may indicate changes in wildlife trade on a broader regional scale.

The coherence of the information obtained through different methodologies, including direct observations and vendor surveys, verified the information, overcoming one of the concerns of studies in markets of wild products in the tropics. Despite general considerations, trends in the trade of tortoises and river turtles should be analyzed separately because their habitats, biology, and trading patterns are different.

We observed a seasonal difference in the sale of river turtles and tortoises. River turtles inhabit rivers, lakes, and channels of flooded forests (*varzea*), and had increased sales during the low-water season (July–September) when harvesting is easier because turtles are concentrated in reduced water bodies and nesting females go onto beaches, where they become easy prey [6,10]. River turtles are also captured in gill nets, often as a by-catch of fishing. Eggs are collected on beaches during the reproductive season. People locate the nests by looking for tracks of turtles in the sand and then use sticks to find soft areas, which disclose the nests.

In contrast, yellow-footed tortoises occur in non-flooded upland forests (*terra firme*) and are collected by hunters opportunistically or by using pit traps baited with rotten meat [20]. The hunting grounds in upland forests are easier to access during the high-water season (December–June) when streams rise and hunters can use canoes to reach far inside the forests [23].

In the early 1980s, river turtles became the subject of increased research and management [6]. In Peru, a head-starting community-based management initiative for turtles, aiming to relocate nests and protect eggs and adults of *P. expansa* and *P. unifilis,* was created in 1994 in the Pacaya-Samiria Natural Reserve (see Figure 1). More recently, a proportion of *P. unifilis* hatchlings from head-started nests have been sold into the legal pet trade in compliance with CITES II regulations, providing Indigenous management groups with income. In the Pacaya Samiria Natural Reserve, there were 32 management groups established by 2010 [33], leading to the recovery of river turtles in the reserve because of the number of river turtles released by the head-starting program [34]. The head-starting program management group focuses on *P. unifilis* and the reserve stations focus on *P. expansa.* Nesting beaches are cleaned before the egg laying season. Nests are collected after females deposit their eggs and are relocated in the same position on a protected artificial beach with similar light conditions. Hatchlings are kept for several days in plastic water tanks, allowing their shells to become wet, and then are released in areas void of their natural predators. There is a high mortality of natural nests during incubation and upon hatching, which makes head-started nests much more successful.

In the Brazilian Amazon, community-based conservation projects have also been effective in increasing and protecting river turtle nests, hatchlings, and stocks of chelonians at a low operational cost [35], while maintaining income generation for the local population [36]. Community-based conservation projects have been restoring river turtle populations since 1974, but these programs began to gain strength between 1990 and 1999, eventually covering 88–94% of chelonian breeding areas in the Central Amazon [35] and promoting an increase in the number of protected nests and hatchlings of Podocnemididae [35,36,37,38,39]. However, it was observed that these populations perhaps represent less than 1–2% of the original population of the 19th century [40]. The success of these programs led to *P. expansa* being removed from the list of endangered species in Brazil in 1996 [38,39]. However, the decrease in the volume of resources devoted to its protection has caused a 15.5–46.8% reduction in the production of hatchlings [41]. This further demonstrates the need for active governmental strategies, combined with community-based programs, to protect river turtles [35].

Our data show a substantial decrease in *P. unifilis* trade (−230.03%) and a halt on *P. expansa* and *P. sextuberculata* sales; at the same time, a large number of river turtle eggs were being sold (570,229 eggs). This might be due to the effect of the aforementioned community-based conservation head-starting programs and the redirection of river turtle trade from meat to eggs. The large numbers of *P. expansa* and *P. unifilis* eggs sold might indicate that the reduction in turtle meat trade is not due to the reduction in wild river turtle populations, but rather to a trade adaptation, focusing on eggs and hatchlings, with a decrease in adult harvesting. Field data recorded on *P. unifilis* in the Pacaya-Samiria Natural Reserve indicate that there has been an increase in the number of eggs sold or consumed, increasing from less than 7000 in 1994 to more than 330,000 in 2009 [33] and 500,000 (very similar to our estimate) in 2020 [19]. Pérez-Peña et al. [42] suggested that this increase may, in part, also be caused by the increase in the number of local groups managing clutches. Since 2022, packaged *P. unifilis* eggs have been legally sold in the large markets of Iquitos, which also show general a focus on eggs, not adults [R. Bodmer, pers. comm.].

Changes in the wild meat market trade can be caused by changes in the trade, or changes in the population of the harvested species. As the focus on the river turtle trade has changed from meat to eggs, monitoring the egg trade could be a better indicator of the wild population than meat sales.

Transforming the number of eggs sold into the number of clutches can estimate the minimum population of wild females. Considering a clutch size between 103 and 106 eggs for *P. expansa* [43,44] and 22 and 35 eggs for *P. unifilis* [6,43,44], our reported sales of 447,089 *P. unifilis* eggs and 123,139 *P. expansa* eggs represent 15,550 and 1178 clutches, respectively, and approximately 10,418 *P. unifilis* and 1178 *P. expansa* reproductive females. These results suggest large populations of river turtles, and that the egg trade in Iquitos is possibly sustainable, and is more sustainable than the meat trade, which can reduce populations of river turtles. Rivera et al. [45] estimated a probability of extinction of *P. unifilis* populations in 30 years of 84% with an increase in juvenile catches from 20% to 40%, and 98% with the removal of 100 adults annually, showing the vulnerability of harvesting for meat. The river turtle egg trade appears to be more profitable than the turtle meat trade for urban vendors, encouraging the protection of adults to increase egg production. Thus, the sale price in urban markets of Iquitos for an average clutch is USD 12.2 for *P. unifilis* and USD 47.0 for *P. expansa*, and is higher than the consumer price of meat (USD 11.3 per adult and USD 15.1, respectively).

We also saw a reduction in the trade of *C. denticulatus* from 17,432 to 7475 individuals. Trade of this species has traditionally been focused on the sale of meat, and not eggs. This species does not retain eggs for a single clutch, but instead spawns continuously [46], burying eggs in the ground or lightly covering them with litter [47,48], making it difficult for local hunters to collect these eggs.

The reduction in the number of *C. denticulatus* adults sold, like river turtles, can be due to changes in harvest pressure or changes in tortoise populations. This tortoise is widely distributed in the Amazon and ranges throughout the *terra firme* forests, covering 250,000 km^2^ in Loreto, whereas river turtles are restricted to rivers, lakes, and channels, which are estimated to cover an area of 38,000 km^2^ across Loreto [49]. Consequently, *C. denticulatus* is only categorized as Vulnerable by the IUCN [2], despite being included in Appendix I of CITES [3] due to its high hunting pressure for subsistence consumption [4,15,29].

In large areas of non-fragmented forest, such as the Amazon, populations of terrestrial species may be more resilient to the impact of hunting due to the existence of adequate spatial refuges [50]. However, we must be cautious, as knowledge on *C. denticulatus* wild populations is still very limited, and the long-term monitoring of populations across the species’ range is needed to assess the sustainability of tortoise harvests.

Since pre-Colombian times, river turtles and tortoises have been important as a food source, and they continue to be important today. The conservation of these chelonians will depend on finding resource-use strategies that help the populations recover. The head-starting program has helped river turtles recover in flooded forests and, at the same time, has provided income for Indigenous management groups through the use of eggs and hatchlings. The harvesting of adults undercuts the head-starting activities and weakens populations, so the observed reduction in the sales of river turtle meat over time is a positive sign. However, there are still large quantities of chelonian meat sold in the Iquitos markets, and this sale is likely not sustainable and requires further conservation efforts.

## 5. Conclusions

The chelonian trade is important for the nutrition, culture, and economy of local people in the Amazon, and the implementation of community-based head-starting has been shown to change the meat and egg trade in urban markets. Our 13-year longitudinal study shows a substantial decline in the chelonian meat trade, although it remains an important economic activity (USD 162M). In the case of the river turtle trade (*P. unifilis* and *P. expansa*), this reduction (or even halt for *P. expansa*) in meat trade was accompanied by an increased trade of eggs of *P. unifilis* and *P. expansa*, with a similar economic turnover (USD 284M). This shows that wild populations of river turtles have not decreased and might be managed sustainably by replacing the meat trade with the egg and hatchling trade. Prioritizing the sale of eggs and hatchlings is a more sustainable harvesting strategy than directly selling adult individuals; however, it is necessary to continue strengthening turtle community-based management programs to relocate nests and protect eggs and adults.

As for *C. denticulatus*, the causes of meat trade reduction are not clear and could indicate either the unsustainable harvest of this species or the replacement of its consumption by other meats. Long-term monitoring of wild tortoise populations is needed to assess the sustainability of their harvests.

## Figures and Tables

**Table 1:** Number of individuals and biomass of chelonians by taxonomic order sold in local markets in Iquitos from 2006 to 2018. Numbers in parentheses are the percentages of each species within each year. The table includes the status conservation of each species according to the IUCN [2], the IUCN Tortoise and Freshwater Turtle Specialist Group (TFTSG) [1]), and CITES [3].

Scientific Name	Common Name	IUCN	TFTSG	CITES Appendix	Reported Information (%)			Estimated Individuals (Biomass)			% Change in Individuals		
Species					2006/07	2014	2017/18	2006/07	2014	2017/18	2006–2014	2014–2018	2006–2018
*Chelonoidis denticulatus*	Yellow-footed tortoise	Vulnerable	Near-Threatened	II	8298.3 (76.7%)	614.7 (99.2%)	2260.0 (85.1%)	17,431.9 (52,295.8)	6598.6 (14,846.9)	7475.4 (22,426.2)	−1.6418	0.1173	−1.3319
*Podocnemis expansa*	Giant Amazon river turtle	Low-risk/Conservation-dependent	Critically Endangered	II	640.9 (5.9%)	0.7 (0.0%)	0.0 (0.0%)	1328.5 (23,912.1)	7.2 (96.6)	0.0 (0.0)	−184.6193	-	-
*Podocnemis sextuberculata*	Six-tubercled Amazon river turtle	Vulnerable	Vulnerable	II	17.0 (0.0%)	0.0 (0.0%)	0.0 (0.0%)	34.9 (52.3)	0.0 (0.0)	0.0 (0.0)	-	-	-
*Podocnemis unifilis*	Yellow-spotted river turtle	Vulnerable	Endangered	II	1863.5 (17.2%)	4.5 (7.3%)	397.0 (14.9%)	3899.0 (13,646.4)	48.3 (169.1)	1181.4 (4123.9)	−79.7092	0.9591	−2.3003
Total					10,819.7	619.8	2657	22,694.2 (89,906.6)	6654.1 (15,112.6)	8656.8 (26,561.1)	−2.4106	0.2313	−1.6216

**Table 2:** Animal protein (total in tons—index of domestic meat and fish consumption per capita (ICPC) in g/inh/day—percentage) in Iquitos in 2006/07, 2014, and 2017/18. “Total” includes domestic meat, fish, meat of wild mammals, and chelonians. Source information: inhabitants and domestic meat and fish [GOREL, personal communication].

Year	2006/07	2014	2017/18
Inhabitants	386,037	432,476	471,993
	(tons–g/inh/day–%)		
Total	19,502.6–138.41–100.00	29,549.3–187.19–100.00	43,746.4–253.93–100.00
Chelonians	89.9–0.64–0.46	15.1–0.09–0.05	26.6–0.15–0.06
Wild meat	323.6–2.30–1.66	327.2–2.07–1.11	442.0–2.57–1.01
Poultry	12,269.0–87.07–62.91	22,946.6–145.37–77.66	34,243.5–198.77–78.28
Buffalo	90.5–0.64–0.46	166.1–1.05–0.56	241.2–1.40–0.55
Ovine	2.5–0.02–0.01	13.4–0.08–0.05	33.5–0.19–0.08
Porcine	697.7–4.95–3.58	2216.4–14.04–7.50	2501.6–14.52–5.72
Bovine	238.0–1.69–1.22	513.1–3.25–1.74	448.1–2.60–1.02
Fish	5791.5–41.10–29.70	3351.4–21.23–11.34	5809.9–33.72–13.28

**Table 3:** Purchase, sale price (in USD/individual), and economic profit (in USD/individual and %) of chelonians traded in local markets in Iquitos in 2017/18. Prices are expressed as average ± standard deviation.

Species	N	Purchased	N	Sale	Profit	% Profit
*P. expansa*	1	15.06	0			
*C. denticulatus*	95	14.59 ± 2.83	23	18.27 ± 3.90	3.67	20.11
*P. unifilis*	31	11.32 ± 3.41	9	21.25 ± 5.56	9.93	46.74
*P. sextuberculata*	0		1	12.05		
Overall	127	13.74 ± 3.30	33	18.77 ± 4.51	5.03	26.78

**Table 4:** Sale price (in USD/portion) of the turtles by anatomical portion, as usually sold in local markets in Iquitos in 2018. Prices are expressed as average ± standard deviation.

	*C. denticulatus*		*P. unifilis*	
Portion	n	X ± SD	n	X ± SD
Hindlimbs	314	8.45 ± 2.11	110	6.48 ± 1.58
Forelimbs	314	7.45 ± 1.94	110	6.17 ± 1.56
Carapace	0		94	3.09 ± 0.80
Liver	282	2.95 ± 0.23	97	2.62 ± 0.60

**Table 5:** Sale prices (in USD per kg) of domestic and wild mammal meat, fish, and chelonians in local markets in Iquitos in 2018.

Species	Sale Prices ± SD (USD/kg)	Difference (%) Compared to Chelonians
*C. denticulatus*	6.09 ± 1.30	
*P. unifilis*	6.07 ± 1.59	
Bovine	4.81 ± 0.23	−20.89
Wild mammal meat	4.47 ± 0.24	−26.48
Porcine	4.36 ± 0.22	−28.29
Total domestic meat	4.10 ± 0.76	−32.57
Poultry	3.14 ± 0.32	−48.36
Fish	3.06 ± 0.17	−49.67

## Data Availability

The data used to support the findings of this study are available from the corresponding author upon reasonable request.

## Supplementary Materials

The following supporting information can be downloaded at: https://www.mdpi.com/article/10.3390/ani14223205/s1, Table S1: Number of individuals by taxonomic order sold monthly in local markets in Iquitos in 2006 and 2018. Table S2: Seasonality of price (in USD/individual) for the purchase of *Chelonoidis denticulata* and *Podocnemis unifilis* bought by regular vendors from intermediaries in local markets in Iquitos, 2017–2018. Table S3: Seasonality and number of turtles purchased by a confident vendor from intermediaries in Iquitos in 2018; price (in USD/egg) for sale of *Podocnemis unifilis* and *Podocnemis expansa* in local markets in Iquitos, 2017–2018. Table S4: Price (in US$ per unit or 100 eggs) for sale of *Podocnemis unifilis* and *Podocnemis expansa*, depending on their preservation method, in the local markets of Iquitos in 2017–2018. Prices are expressed in US per unit or 100 eggs, because the price difference between sales to retailers and wholesalers is considerable. Figure S1: Seasonality of the number of turtles purchased by a confident vendor from intermediaries in Iquitos and the Amazon River level (m.a.s.l.) during 2017 and 2018.
