# Supplementary material for: Trends in Urban Wild Meat Trade of Chelonians (Turtles and Tortoises) in the Peruvian Amazon
Source: Animals (Basel). 2024 Nov 8;14(22):3205. doi: 10.3390/ani14223205 (PMC11591361; doi:10.3390/ani14223205)

Suppl. Table S1. Number of individuals by taxonomic order monthly sold in local markets in Iquitos in 2006 and 2018. All the individuals sold in each month have been grouped by adding the two 12-month surveys. Numbers in parentheses are the individuals sold per day.

Species	Jan	Feb	March	Apr	May	June	July	Ag	Sept	Oct	Nov	Dec	Total (%)
Chelonoidis denticulata	897 (18.31)	1131 (25.13)	1355 (28.23)	1028 (23.35)	981.5 (20.45)	784.3 (16.69)	736 (17.52)	639 (14.20)	707.5 (21.44)	1025 (22.28)	967 (25.45)	768.5 (18.74)	11019 (75.05)
Podocnemis expansa	3 (0.06)	- (-)	- (-)	- (-)	2 (0.04)	10 (0.21)	54 (1.29)	119 (2.64)	128 (3.88)	18 (0.39)	50 (1.32)	22 (0.54)	406 (2.77)
Podocnemis sextuberculata	- (-)	- (-)	- (-)	- (-)	- (-)	- (-)	14 (0.33)	8 (0.18)	- (-)	- (-)	- (-)	12 (0.29)	34 (0.23)
Podocnemis unifilis	12 (0.24)	20 (0.44)	57 (1.19)	28 (6.36)	175 (3.65)	504 (10.72)	665 (15.83)	1024 (22.76)	144 (4.36)	230 (5.00)	209 (5.50)	156 (3.80)	3224 (21.96)
TOTAL	912 (18.61)	1151 (25.58)	1412 (29.42)	1056 (23.99)	1159 (24.14)	1298 (27.62)	1469 (34.98)	1790 (39.78)	979.5 (29.68)	1273 (27.67)	1226 (32.26)	958.5 (32.26)	14683 (100.00)

Suppl. Table S2. Seasonality of the price (in US$/individual) for the purchase of *Chelonoidis denticulata* and *Podocnemis unifilis* bought by regular vendors to intermediaries in local markets in Iquitos 2017-2018.

Month	*Chelonoidis denticulata*		*Podocnemis unifilis*	
	N	X ± SD	N	X ± SD
Jan	317	15.1 ± 1.8	0	
Feb	160	14.1 ± 0.9	0	
March	190	11.8 ± 1.2	0	
April	80	12.0 ± 1.6	14	9.8 ± 3.2
May	69	13.6 ± 4.0	14	14.1 ± 1.7
June	114	17.4 ± 1.9	74	14.3 ± 1.5
July	10	22.6 ± 1.5	5	24.1 ± 12.8
Aug	17	15.4 ± 7.8	19	8.3 ± 2.9
Sept	177	14.3 ± 2.8	12	13.6 ± 2.5
Oct	139	12.9 ± 2.2	21	8.6 ± 2.1
Nov	71	15.3 ± 3.8	8	12.8 ± 5.1
Dec	260	17.0 ± 2.6	0	
Total	1604		167	

Suppl. Table S3. Seasonality and number of turtles purchased by a confident vendor from intermediaries in Iquitos in 2017-2018.

Species	Jan	Feb	March	Apr	May	June	July	Aug	Sept	Oct	Nov	Dec	Total
Cheloinidis denticulata	73	118	200	80	65	139	26	31	58	100	76	78	1044
Podocnemis unifilis	0	0	0	0	0	0	150	170	8	8	0	0	336
TOTAL	73	118	200	80	65	139	176	201	66	108	76	78	1380

Suppl. Table S4. Price (in US$ per unit or 100 eggs) for sale of *Podocnemis unifilis* and *Podocnemis expansa*, depending on their preservation method, in the local markets of Iquitos in 2017-2018. Prices are expressed in US per unit or 100 eggs, because the price difference between sales to retailers and wholesalers is considerable.

Species	Preservation	N	Price (US/egg)	N	Price (US/100 eggs)
P. expansa	Cooked	130	0.47 (SD0.14)	31	28.76 (SD2.66)
	Raw salty	40	0.40 (SD0.13)		
	Subtotal	170	0.45 (SD0.14)	32	28.71 (SD2.63)
P. unifilis	Cooked	290	0.45 (SD0.14)	130	27.27 (SD3.48)
	Fresh	2	0.60 (SD0.10)	3	30.12 (SD0.00)
	Raw salty	118	0.42 (SD0.13)	25	28.43 (SD2.76)
	Subtotal	410	0.44 (SD0.13)	185	28.89 (SD3.26)
	Total	580	0.45 (SD0.14)	217	28.02 (SD3.18)

Suppl. Figure S1. Seasonality of the number of turtles purchased by a confident vendor from intermediaries in Iquitos, and the Amazon River level (m.a.s.l.) during 2017 and 2018.

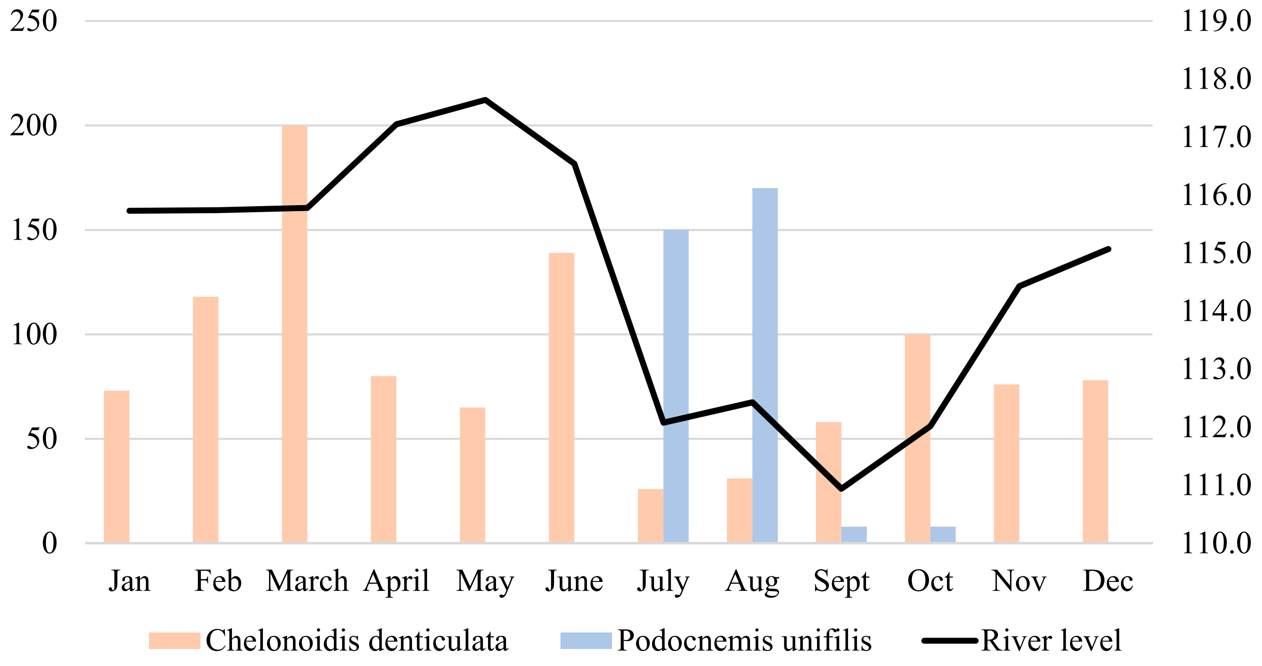

Supplement: Supplementary file 1 [file animals-14-03205-s001.zip › animals-3216985-supplementary.pdf]
